# Intrafraction Prostate Motion Management for Ultra-Hypofractionated Radiotherapy of Prostate Cancer

**DOI:** 10.3390/curroncol29090496

**Published:** 2022-08-31

**Authors:** Christoph Oehler, Nina Roehner, Marcin Sumila, Jürgen Curschmann, Fabrizio Storelli, Daniel Rudolf Zwahlen, Uwe Schneider

**Affiliations:** 1 Department of Radiation Oncology, Hospital Winterthur, 8400 Winterthur, Switzerland; 2 Department of Radiation Oncology, Hirslanden Zurich, 8032 Zurich, Switzerland or

**Keywords:** prostate cancer, prostate motion, radiation therapy, ultra-hypofractionation, stereotactic, IGRT, radiopaque fiducial marker, tracking, PTV margin, MR-based adaptive radiotherapy

## Abstract

Purpose: Determine the time-dependent magnitude of intrafraction prostate displacement and a cutoff for the tracking decision. Methods: Nine patients with localized prostate cancer were treated with ultra-hypofractionated radiotherapy (CyberKnife) with fiducial markers. Exact tract kV/kV imaging was used with an average interval of 19–92 s. A Gaussian distribution was calculated for the *x*-, *y*-, and *z*-directions (*σ_x,y,z_*). The variation of prostate motion (*μ_σ_*) was obtained by averaging the patients’ specifics, and the safety margin was calculated to be MAB = WYm + WBSs. Results: The calculated PTV safety margins were as follows: at 40 s: 0.55 mm (L/r), 0.85 mm (a/p), and 1.05 mm (s/i); at 60 s: 0.9 mm (L/r), 1.35 mm (a/p), and 1.55 mm (s/i); at 100 s: 1.5 mm (L/r), 2.3 mm (a/p), and 2.6 mm (s/i); at 150 s: 1.9 mm (L/r), 3.1 mm (a/p), and 3.6 mm (s/i); at 200 s: 2.2 mm (L/r), 3.8 mm (a/p), and 4.2 mm (s/i); and at 300 s: 2.6 mm (L/r), 5.3 mm (a/p), and 5.6 mm (s/i). A tracking cutoff of 2.5 min seemed reasonable. In order to achieve an accuracy of < 1 mm, tracking with < 50 s intervals was necessary. Conclusions: For ultra-hypofractionated radiotherapy of the prostate with treatment times > 2.5 min, intrafraction motion management is recommended.

## 1. Introduction

Prostate cancer is the most common cancer in men, with an incidence of 113/100,000 men per year [1]. Radiotherapy (RT) is one of the main curative treatment modalities besides surgery. Recently, ultra-hypofractionated radiotherapy has been proven to be noninferior to conventionally fractionated radiotherapy, and it has been increasingly employed since [2].

The use of ultra-hypofractionated radiotherapy leads to prolonged treatment times compared with normal fractionated and moderately hypofractionated RT. Treatment duration may vary substantially from 1 min to more than 10 min and depends not only on the fractionation scheme but also on the treatment machine (linear accelerator (LINAC) vs. CyberKnife) or beam modulation (flattening filter-free (FFF) vs. flattening filter) [3,4]. 

Treatment duration plays a crucial role in the amount of intrafraction prostate motion [3,5]. Prolonged treatment time of 10 min compared with the 5 min one leads to increased prostate shifts by 3 mm and more [6,7,8]. Consecutively, prolonged treatment times harbor the risk of geographical miss and demand either the adoption of larger planned target volume (PTV) margins at the cost of normal tissue exposure and toxicity or intrafraction tracking/monitoring. For moderately hypofractionated RT, intrafraction prostate motion is usually compensated for with the adaptation of the PTV margin [9]. On the other hand, for stereotactic and ultra-hypofractionated RT, prostate tracking/monitoring is the preferred method. Currently, it is not clear which treatment times demand intrafraction prostate motion tracking rather than compensation with safety margins.

Various techniques have been proposed for prostate motion monitoring and compensation, including intraprostatic radiopaque fiducial markers (FMs) with in-room imaging, ultrasound-based systems, electromagnetic tracking, and MRI systems integrated into the treatment room, all of which are suitable for LINACs [10]. For CyberKnife treatment, the use of intraprostatic radiopaque FMs is required. Radiopaque markers require repetitive intrafraction imaging. Some institutions investigated an imaging interval of 40 s [7] or 60–180 s [6], while others suggested an imaging frequency depending on margin size, i.e., every 15, 60, or 240 s for 1 mm, 2 mm, or 3 mm margins, respectively [11]. Currently, there are no guidelines on what imaging frequency should be used. 

This study aimed to use intraprostatic radiopaque FM tracking by repetitive in-room imaging in order to (1) evaluate the time-dependent magnitude of intrafraction translational prostate displacement, (2) determine an optimal intrafraction imaging frequency allowing minimal margins, and (3) determine a time cutoff for decision-making regarding the compensation modality, i.e., PTV margins compensation vs. intrafraction prostate tracking.

## 2. Materials and Methods

### 2.1. Study Design and Treatment

Radiotherapy treatment data of nine patients with localized prostate cancer treated with ultra-hypofractionated stereotactic RT (5 × 7.25 Gy) using a CyberKnife linear accelerator at a single institution in Switzerland between 05/2009 and 11/2012 were retrospectively analyzed. Informed consent was obtained before treatment. Patient positioning was the same for the patients treated with a LINAC: the patients were treated in supine position with comfortable bladder filling and emptied rectum using laxatives but without an endorectal balloon (ERB). For IGRT, radiopaque fiducial markers were implanted into the prostate before planning CT, and their position was tracked during treatment using a kV/kV orthogonal imaging system (integrated kV/kV system). The treatment time was between 1500 and 6000 s. Imaging was performed with an average interval of 19–92 s during treatment. FM position data were immediately analyzed, and displacement data were sent to the robotic system, allowing the robotic arm to follow the target. No table adjustments were performed.

### 2.2. Data Analysis

The prostate position was recorded in the *x*-, *y*-, and *z*-directions. Differences in the *x*-axis correspond to left/right movements, in the *y*-axis—to anterior/posterior movements, and in the *z*-axis—to inferior/superior movements (DICOM standard). Neither rotational nor deformational movements were analyzed. Prostate position data were recorded in a log file during treatment. For data analysis, programming language IDL (Version 8.7 interactive data language) was used. The log files created during treatment were imported into the IDL code to be evaluated statistically. 

### 2.3. Statistics

CTV_prostate_ displacement patterns were graphically depicted as a function of timeout of the log file for each patient and all the treatment sessions in all three spatial directions by connecting data of the fractions to one total pattern. CTV_prostate_ displacement patterns were evaluated for time intervals I of 0 s, 50 s, 100 s, 150 s, 200 s, and 300 s. Additionally, the displacement after each imaging section was assessed to calculate the necessary PTV safety margin for CyberKnife treatment. Since all the data were discrete and did not match the above time intervals, the closest datapoint was chosen. Therefore, the actual average time interval steps were I = 41.9 s (nominal, 0 s), 56.9 s (nominal, 50 s), 101 s (nominal, 100 s), 150.8 s (nominal, 150 s), 200.4 s (nominal, 200 s), and 299.4 s (nominal, 300 s). CTV_prostate_ displacement was graphically depicted with frequency histograms with bin size 0.2 mm created for each piece of patient data and for each of the time intervals. 

Assuming the prostate motion was random with a Gaussian distribution, a “propensity density function” (Equation (1)) was fitted for the *x*-, *y*-, and *z*-directions (*σ_x,y,z_*).

(1) $$f\left(x,\sigma_{x}\right)=\frac{1}{\sigma_{x}\sqrt{2\pi }}exp^{-\frac{{\left(x-\mu_{x}\right)}^{2}}{2\sigma_{x}^{2}}}$$

This function describes a symmetric curve centered on the mean *μ_x_* with the total area under the curve equal to 1. The values *σ_x,y,z_* give the standard deviation of the distribution in all directions. CTV_prostate_ motion could be quantified by the variation of *σ_x,y,z_*, which is proportional to the probability that the CTV_prostate_ lies within the PTV. The proportionality constants that correspond to a certain coverage of the PTV are listed in Table 1. 

The variation of the CTV_prostate_ movements (*μσ*) in the patient cohort was called *μ^j^*_Φ073_ and was obtained by averaging the patients’ specific *σ^j^_i_*, and *j* specified the spatial direction. The standard deviation ∑*^j^* of this average corresponded to the patient variation. 

The PTV safety margin into the *j* direction valid for A% of all the patients for whom the CTV_prostate_ lies with a probability B% into the PTV was calculated as follows:

(2) $$M_{AB}^{j}=W_{A}\mu_{\sigma }^{j}+W_{B}\Sigma_{\sigma }^{j}$$

## 3. Results

### 3.1. Prostate Displacement Pattern

**Figure 1:**
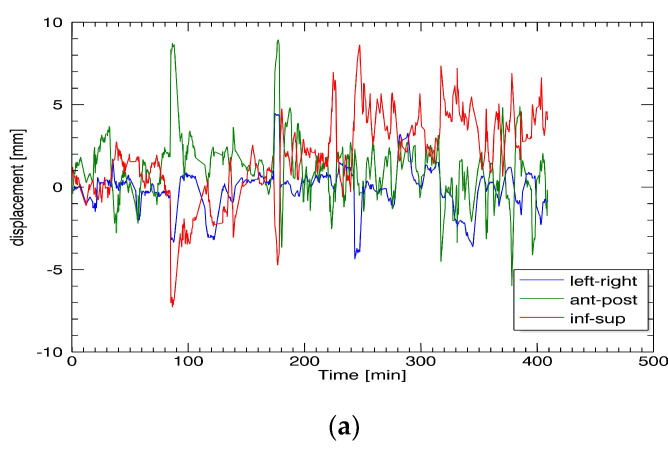

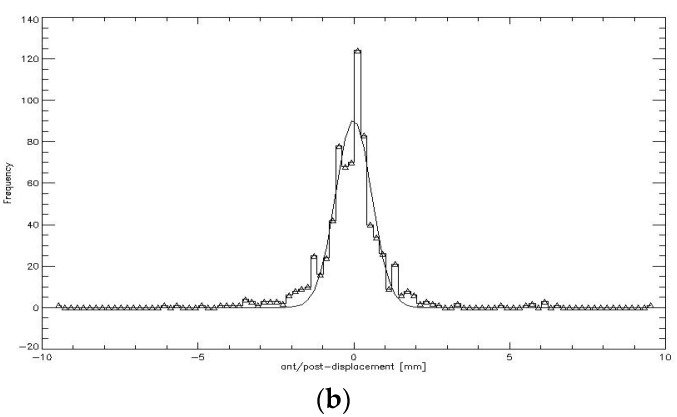
CTV_prostate_ displacement pattern of one patient in all directions, *x*, *y*, and *z* (**a**), and distribution of the CTV_prostate_ in one patient in anterior/posterior direction using a time interval of I = 50 s (**b**).

First, we graphically displayed the CTV_prostate_ displacement pattern as a function of the time of all the treatment sessions (Figure 1a). The maximum displacement in any direction did not exceed 10 mm. The CTV_prostate_ displacement was a Gaussian distribution (Figure 1b). The mean values were close to 0 and an order of magnitude smaller than the standard deviations, meaning the systematic errors could be neglected due to FM-guided displacement corrections during treatment.

### 3.2. Imaging Interval and Internal Margin

We next calculated the safety margins using the time length intervals of I = 0, 50, 100, 150, 200, and 300 s (Table 2, Table 3, Table 4, Table 5, Table 6 and Table 7). 

**Figure 2:**
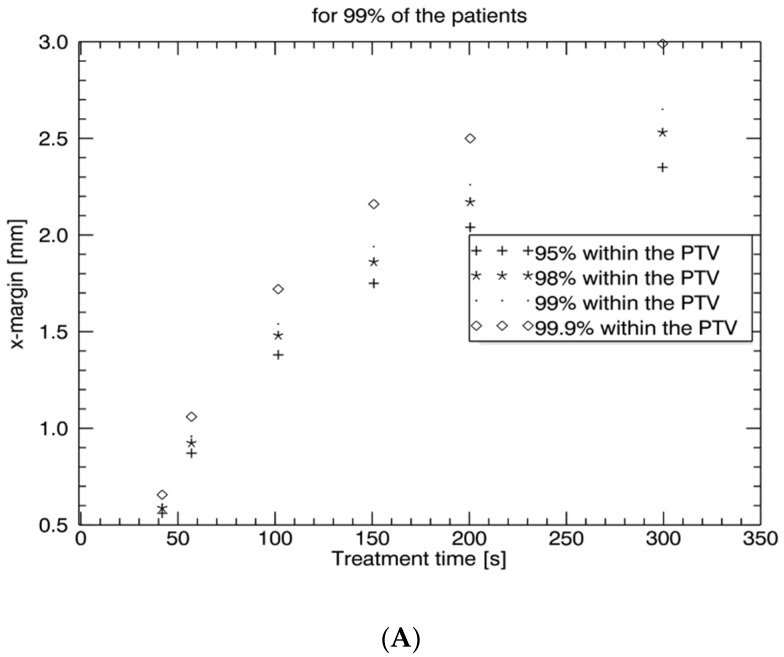

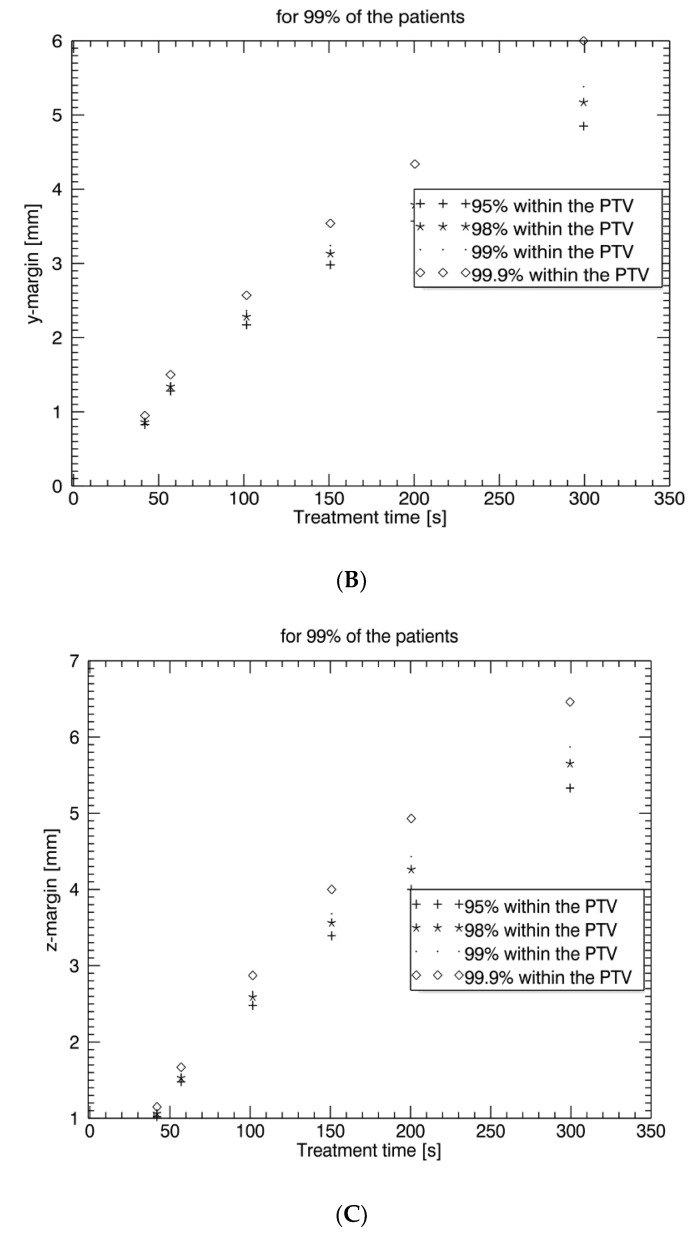
PTV safety margin curves dependent on time interval I = 50–300 s with a 95%, 98%, 99%, and 99.9% probability (for 99% of the patients) in the x-direction (left/right) (**A**), the y-direction (anterior/posterior) (**B**), and the z-direction (inferior/superior) (**C**).

The PTV safety margins in all three directions, *x*, *y*, and *z*, for 95%, 98%, 99%, and 99.9% coverage probability for 99% of the patients are depicted in Figure 2A–C. To treat the CTV_prostate_ with an accuracy of <1 mm in all the directions, tracking with less than 50-s intervals was necessary.

### 3.3. PTV Safety Margins

**Figure 3:**
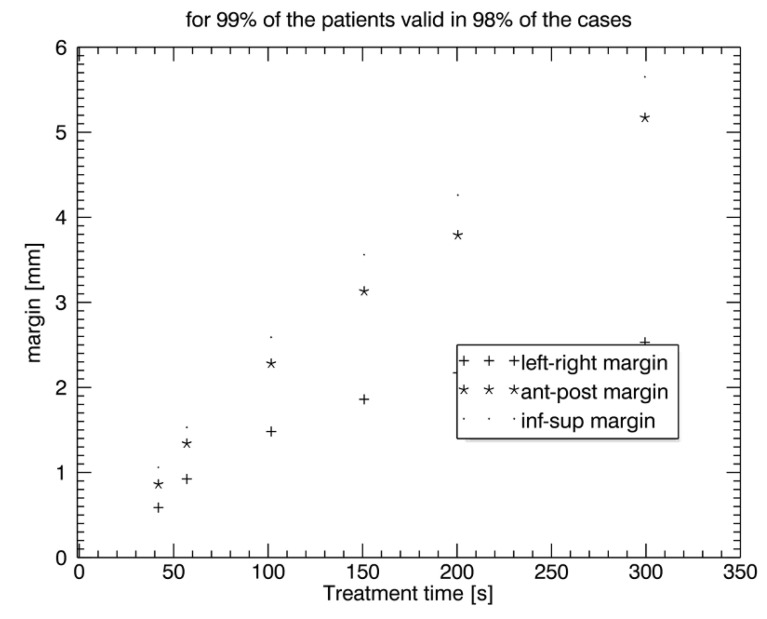
Comparison of the PTV safety margins in all three directions, *x*, *y*, and *z*, with an increasing time interval with 98% probability in 99% of the patients.

Figure 3 shows a comparison of PTV safety margins with 98% coverage probability (for 99% of patients) for all three directions, *x*, *y*, and *z*. PTV safety margins were similarly large for *y*- and *z*-directions, whereas they were clearly smaller for the *x*-direction (left/right), particularly with an increasing time interval. With CTV_prostate_ tracking with a time interval below 50 s, PTV safety margins appeared to be 0.5 mm–1 mm in all three directions, *x*, *y*, and *z*. Since intrafraction movement during 5 min of treatment time could be as high as 2.5–5–6 mm, intrafraction tracking allowed PTV margins reduction by 2 (left–right)–4.5 mm (anterior–posterior/superior–inferior) depending on the geographical axis (Figure 3).

PTV margins for 99% of the patients with 98% coverage probability were as follows: at 40 s: 0.55 mm (L/r), 0.85 mm (a/p), 1.05 mm (s/i); at 60 s: 0.9 mm (L/r), 1.35 mm (a/p), 1.55 mm (s/i); at 100 s: 1.5 mm (L/r), 2.3 mm (a/p), 2.6 mm (s/i); at 150 s: 1.9 mm (L/r), 3.1 mm (a/p), 3.6 mm (s/i); at 200 s: 2.2 mm (L/r), 3.8 mm (a/p), 4.2 mm (s/i); at 300 s: 2.6 mm (L/r), 5.3 mm (a/p), 5.6 mm (s/i).

In summary, CTV_prostate_ tracking with time intervals below 50 s reduced intrafraction errors to 0.5–1 mm in all directions.

## 4. Discussion

Ultra-hypofractionated and stereotactic radiotherapy and adaptive radiotherapy result in prolonged treatment times. Radiation treatment time plays a crucial role in the magnitude of intrafraction prostate motion [5]. Therefore, intrafraction motion management is important in these settings to avoid geographical miss and normal tissue exposure. Our study confirms that intrafraction uncertainty is time-dependent. For treatment times below 2.5 min (<150 s), which are generally needed for moderately hypofractionated radiotherapy with conventional LINACs, intrafraction prostate motion is low and may be reasonably compensated by a PTV margin of an additional 2–3 mm. However, for treatment times above 2.5 min, as generally needed for stereotactic radiotherapy and ultra-hypofractionation without an FF, intrafraction monitoring and correction are recommended since prostate dislocations are unacceptably high (5 min: > 5 mm). Our study further shows that an intrafraction imaging interval < 50 s with consecutive prostate tracking enables PTV safety margins for intrafraction uncertainty between 0.5 mm and 1 mm in all three directions, *x*, *y*, and *z*. Due to the low number of included patients, the statistical results of this study need to be seen in consideration of other studies.

The duration of radiotherapy may vary substantially from 1 min to more than 10 min depending on the fractionation scheme, treatment machine (CyberKnife vs. LINAC), and beam modulation (FFF vs. flattening filter) [3,4]. Currently, with the evolution of adaptive radiotherapy systems (e.g., Ethos, Varian Medical Systems, Palo Alto, CA, USA; MR–LINAC hybrid systems), treatment times are further increased by at least 213 s [12]. Studies have shown that prostate motion > 2 mm occurs in about 5% of the cases at 30 s, 8%—at 60 s, 11%—at 90 s, 14%—at 120 s [7,13,14]. Prolonged RT times can increase intrafraction prostate movement from 3 up to 7 mm, which equals the margin from the CTV to the PTV [15,16]. With a median treatment time of 16 min, intrafraction shifts of the prostate > 5 mm were found in 12% of all the fractions, and a margin of 6 mm was calculated for compensation for this uncertainty [15]. Mobility of the prostate weakly correlated with changes in the rectal volume but was independent of the bony anatomy or intrafraction bladder re-filling by 41 ccm [15]. Other groups investigating intrafraction prostate motion with LINACs or rotational radiotherapy systems (e.g., Tomotherapy, Accuray, Sunnyvale, CA, USA) reported an overall error margin of 2–3 mm but did not indicate time dependency [17,18,19]. Our study confirms that prostate shifts are time-dependent and depicts in detail rising correction margins of 0.5–1 mm at 40 s, 0.9–1.55 mm at 60 s, 1.5–2.6 mm at 100 s, 1.9–3.6 mm at 150 s, 2.2–4.2 mm at 200 s, and 2.6–5.6 mm at 300 s. 

Currently, different methods for prostate motion management exist: (1) implanted radiopaque (gold) FM and kV/kV imaging for automatic three- to six-dimensional intrafraction prostate motion corrections (e.g., ExacTrac, Brainlab, Munich, Germany, as used in this study; Truebeam Auto Beam Hold^®^ tracking system, Varian Medical Systems, USA); (2) clarity autoscan with a monitoring system and its use of noninvasive soft tissue imaging to monitor prostate motion during the course of radiotherapy; this system uses a 4D autoscan ultrasound probe to image the prostate through the acoustic window of the perineum (Elekta Instrument AB, Stockholm, Sweden) [20]; (3) implanted radiofrequency transponder beacons (Calypso system, Varian Medical Systems, Palo Alto, CA, USA); and more recently, (4) MRI-integrated systems (MRIdian, ViewRay, Oakwood Village, OH, USA; Electa Unity, Elekta Instrument AB, Stockholm, Sweden). Each technology is suitable for high-precision RT. Localization of the prostate using electromagnetic transponders agreed well with optical radiographic techniques [21,22]. However, systems using real-time adaptation were reported to significantly outperform nonadaptive delivery methods [23]. The mean 2%/2 mm γ-fail rate was 1.4% with adaptation but 17.3% without adaptation in prostate treatment (*p* < 0.001) [23]. Some argue that real-time tracking is necessary since respiratory-induced prostate mobility within 30 s during radiotherapy is significant, especially in the superior–inferior direction, and can thus induce a geographical error [13,14]. Real-time couch-tracking resulted in submillimeter accuracy for prostate cancer, which transferred into high dosimetric accuracy [24]. However, the mean displacements were minimal: 1.4 mm (–3.1 to 8.2 mm), –2.2 mm (–9.1 to 1.5 mm), and –0.3 mm (–5.0 to 1.8 mm) in the AP, SI, and LR directions, respectively [13,14]. A study by the MSKCC using implanted radiofrequency transponder beacons found that after the initial setup, 1.7 interventions per fraction were required to keep the prostate within 2 mm of its planned position, with a concomitant increase in time for dose delivery of approximately 65 s [25]. The posterior PTV margin required for 95% of the dose to be delivered with the target positioned within the PTV was found to increase by 2 mm every 5 min [25]. Our study shows that for treatment times below 2.5 min (<150 s), intrafraction prostate motion may be reasonably compensated by an additional PTV margin of 2–3 mm. However, for treatment times above 2.5 min, intrafraction monitoring and correction are recommended with an imaging interval of <50 s. This allows for PTV safety margins for intrafraction uncertainty between 0.5 mm and 1 mm. The dosimetric impact of online translation corrections based on the FM positions in kV/kV was analyzed using cine-MRI within the HypoFLAME trial, which investigated ultra-hypofractionated RT (5 × 7 Gy; average treatment time = 6 min) [26]. They found that an overall PTV margin of 4–5 mm and online translation correction resulted in D99% coverage in 97% (CTV_prostate_) and 100% (CTV_seminal vesicles_) of the cases, whereas D99% for GTV_boost_ was only 87% [26]. 

Treatment time for SBRT can be significantly reduced by using the FFF mode [27]. Several articles reported significantly faster beam-on time (BOT) and treatment time (T × T) for FFF plans compared with flattened beam (FB) plans. The planning doses across these studies were 5–25 Gy per fraction. The mean BOT across the three studies for FB was 6.39 min, for FFF—2.42 min. The mean T × T was 11.21 min and 5.79 min for FB and FFF, respectively. Another method of reducing prostate motion for prostate SBRT is using endorectal immobilization with a rectal displacement device (RDD) [28]. The RDD used in prostate SBRT leads to reduced intrafraction motion of the prostate and rectum, with increasing improvement with time. It also results in significant improvement in rectal wall dosimetry. Furthermore, Timmerman et al. showed that prostate immobilization with endorectal balloon (ERB) interventions reduced prostate deformation in the anterior–posterior direction, reduced errors in the sagittal rotation of the prostate, and increased the similarity in the shape of the prostate to the radiotherapy plan and resulted in improved dose–volume histogram (DVH) characteristics [29]. Another group compared ERB interventions with a hydrogel spacer and showed comparable prostate motion between an ERB and a hydrogel spacer [30]. The time dependencies were similar. A large majority of shifts for both ERB and hydrogel were well within a typical robust planning margin.

The evolution of adaptive radiotherapy (ART) inevitably results in further prolonged treatment preparation times in addition to beam-on times [12,31]. In the clinical setting, MR-based ART required an average of 45 min per fraction [31]. Newer artificial intelligence (AI)-based ART systems might shorten the adaptation process. Nevertheless, optimized methods resulting in plans meeting the constraints required at least 213 s for the shape adaptation workflow [12]. During the treatment–preparation time of 213 s, prostate shifts would require PTV margins of more than 4 mm, according to our results. Hence, MR or CBCT (cone-beam computed tomography) reimaging and target realignment before the start of the actual treatment is reasonable, irrespective of the intrafraction compensation method.

## 5. Conclusions

Intrafraction motion management is recommended for ultra-hypofractionated RT of the prostate with treatment times above 2.5 min, including monitoring and correction, since prostate dislocations are unacceptably high. An intrafraction imaging interval of < 50 s allows PTV safety margins for intrafraction uncertainties between 0.5 and 1 mm. Intrafraction prostate motion is low for treatment times below 2.5 min and may be reasonably compensated by an additional PTV margin of 2–3 mm. Reimaging and target realignment may be recommended for extremely prolonged treatment durations, as with AI- and MR-based ART.

## Figures and Tables

**Table 1:** Proportionality constants that correspond to a certain coverage of the PTV.

Percentage (A, B)	95%	98%	99%	99.9%
Weighting factors (W_A_, W_B_)	1.96	2.33	2.58	3.29

**Table 2:** Safety margins using the time length intervals I = 0 s.

% Patients *A*	Direction *j*	$W_{A}\mu_{\sigma }^{j}$ [mm]	$\Sigma_{\sigma }^{j}\;$[mm]	$M_{A95}^{j}\;$[mm]	$M_{A98}^{j}\;$[mm]	$M_{A99}^{j}\;$[mm]	$M_{A99.9}^{j}\;$[mm]
95	*x*	0.319	0.0719	0.460	0.487	0.505	0.556
	*y*	0.493	0.0914	0.672	0.706	0.729	0.794
	*z*	0.623	0.102	0.823	0.861	0.886	0.959
98	*x*	0.379	0.0719	0.520	0.547	0.565	0.616
	*y*	0.586	0.0914	0.765	0.799	0.822	0.887
	*z*	0.740	0.102	0.940	0.978	1.000	1.080
99	*x*	0.419	0.0719	0.56	0.587	0.605	0.656
	*y*	0.648	0.0914	0.827	0.861	0.884	0.949
	*z*	0.818	0.1020	1.020	1.060	1.080	1.150
99.9	*x*	0.536	0.0719	0.677	0.704	0.722	0.773
	*y*	0.828	0.0914	1.010	1.040	1.060	1.130
	*z*	1.050	0.1020	1.250	1.290	1.310	1.390

**Table 3:** Safety margins using the time length intervals I = 50 s.

% Patients *A*	Direction *j*	$W_{A}\mu_{\sigma }^{j}$ [mm]	$\Sigma_{\sigma }^{j}\;$[mm]	$M_{A95}^{j}\;$[mm]	$M_{A98}^{j}\;$[mm]	$M_{A99}^{j}\;$[mm]	$M_{A99.9}^{j}\;$[mm]
95	*x*	0.454	0.140	0.728	0.780	0.815	0.915
	*y*	0.723	0.167	1.050	1.110	1.150	1.270
	*z*	0.898	0.150	1.190	1.250	1.290	1.390
98	*x*	0.540	0.140	0.814	0.866	0.901	1.000
	*y*	0.859	0.167	1.190	1.250	1.290	1.410
	*z*	1.070	0.150	1.360	1.420	1.460	1.560
99	*x*	0.597	0.14	0.871	0.923	0.958	1.060
	*y*	0.950	0.167	1.280	1.340	1.380	1.50
	*z*	1.180	0.150	1.470	1.530	1.570	1.670
99.9	*x*	0.763	0.14	1.090	1.120	1.220	
	*y*	1.210	0.167	1.540	1.600	1.640	1.760
	*z*	1.510	0.150	1.800	1.860	1.900	2.000

**Table 4:** Safety margins using the time length intervals I = 100 s.

% Patients *A*	Direction *j*	$W_{A}\mu_{\sigma }^{j}$ [mm]	$\Sigma_{\sigma }^{j}\;$[mm]	$M_{A95}^{j}\;$[mm]	$M_{A98}^{j}\;$[mm]	$M_{A99}^{j}\;$[mm]	$M_{A99.9}^{j}\;$[mm]
95	*x*	0.677	0.252	1.17	1.26	1.33	1.51
	*y*	1.210	0.302	1.80	1.91	2.00	2.20
	*z*	1.440	0.299	2.03	2.14	2.21	2.42
98	*x*	0.805	0.252	1.30	1.39	1.46	1.63
	*y*	1.430	0.302	2.02	2.13	2.21	2.42
	*z*	1.710	0.299	2.30	2.41	2.48	2.69
99	*x*	0.890	0.252	1.38	1.48	1.54	1.72
	*y*	1.580	0.302	2.17	2.28	2.36	2.57
	*z*	1.890	0.299	2.48	2.59	2.66	2.87
99.9	*x*	1.140	0.252	1.64	1.73	1.79	1.97
	*y*	2.020	0.302	2.61	2.72	2.80	3.01
	*z*	2.420	0.299	3.01	3.12	3.19	3.40

**Table 5:** Safety margins using the time length intervals I = 150 s.

% Patients *A*	Direction *j*	$W_{A}\mu_{\sigma }^{j}$ [mm]	$\Sigma_{\sigma }^{j}\;$[mm]	$M_{A95}^{j}\;$[mm]	$M_{A98}^{j}\;$[mm]	$M_{A99}^{j}\;$[mm]	$M_{A99.9}^{j}\;$[mm]
95	*x*	0.873	0.306	1.47	1.59	1.66	1.88
	*y*	1.640	0.422	2.47	2.62	2.73	3.03
	*z*	1.890	0.460	2.79	2.96	3.08	3.40
98	*x*	1.040	0.306	1.64	1.75	1.83	2.05
	*y*	1.950	0.422	2.78	2.93	3.04	3.34
	*z*	2.250	0.460	3.15	3.32	3.44	3.76
99	*x*	1.150	0.306	1.75	1.86	1.94	2.16
	*y*	2.150	0.422	2.98	3.13	3.24	3.54
	*z*	2.490	0.460	3.39	3.56	3.68	4.00
99.9	*x*	1.470	0.306	2.07	2.18	2.26	2.48
	*y*	2.750	0.422	3.58	3.73	3.84	4.14
	*z*	3.180	0.460	4.08	4.25	4.37	4.69

**Table 6:** Safety margins using the time length intervals I = 200 s.

% Patients *A*	Direction *j*	$W_{A}\mu_{\sigma }^{j}$ [mm]	$\Sigma_{\sigma }^{j}\;$[mm]	$M_{A95}^{j}\;$[mm]	$M_{A98}^{j}\;$[mm]	$M_{A99}^{j}\;$[mm]	$M_{A99.9}^{j}\;$[mm]
95	*x*	1.04	0.344	1.71	1.84	1.93	2.17
	*y*	1.85	0.582	2.99	3.21	3.35	3.76
	*z*	2.00	0.703	3.38	3.64	3.81	4.31
98	*x*	1.24	0.344	1.91	2.04	2.13	2.37
	*y*	2.19	0.582	3.33	3.55	3.69	4.10
	*z*	2.37	0.703	3.75	4.01	4.18	4.68
99	*x*	1.37	0.344	2.04	2.17	2.26	2.50
	*y*	2.43	0.582	3.57	3.79	3.93	4.34
	*z*	2.62	0.703	4.000	4.26	4.43	4.93
99.9	*x*	1.75	0.344	2.4	2.55	2.64	2.88
	*y*	3.10	0.582	4.24	4.46	4.60	5.01
	*z*	3.35	0.703	4.73	4.99	5.16	5.66

**Table 7:** Safety margins using the time length intervals I = 300 s.

% Patients *A*	Direction *j*	$W_{A}\mu_{\sigma }^{j}$ [mm]	$\Sigma_{\sigma }^{j}\;$[mm]	$M_{A95}^{j}\;$[mm]	$M_{A98}^{j}\;$[mm]	$M_{A99}^{j}\;$[mm]	$M_{A99.9}^{j}\;$[mm]
95	*x*	1.08	0.476	2.01	2.19	2.31	2.65
	*y*	2.39	0.870	4.10	4.42	4.63	5.25
	*z*	2.76	0.868	4.46	4.78	5.00	5.62
98	*x*	1.28	0.476	2.21	2.39	2.51	2.85
	*y*	2.84	0.870	4.55	4.87	5.08	5.70
	*z*	3.28	0.868	4.98	5.30	5.52	6.14
99	*x*	1.42	0.476	2.35	2.53	2.65	2.99
	*y*	3.14	0.870	4.85	5.17	5.38	6.00
	*z*	3.63	0.868	5.33	5.65	5.87	6.49
99.9	*x*	1.81	0.476	2.74	2.92	3.04	3.38
	*y*	4.01	0.870	5.72	6.04	6.25	6.87
	*z*	4.64	0.868	6.34	6.66	6.88	7.50

## Data Availability

The data are contained within the article.

## References

[1] NIH (2022). Cancer Stat Facts: Prostate Cancer. https://seer.cancer.gov/statfacts/html/prost.html.

[2] Widmark A., Gunnlaugsson A. (2019). Ultra-hypofractionated versus conventionally fractionated radiotherapy for prostate cancer: 5-year outcomes of the HYPO-RT-PC randomised, non-inferiority, phase 3 trial. Lancet.

[3] Ballhausen H., Li M. (2018). Shorter treatment times reduce the impact of intra-fractional motion: A real-time 4DUS study comparing VMAT vs. step-and-shoot IMRT for prostate cancer. Strahlenther. Onkol..

[4] Benedek H., Lerner M. (2018). The effect of prostate Motion during hypofractionated radiotherapy can be reduced by using flattening filter free beams. Phys. Imaging Radiat. Oncol..

[5] Li J.S., Lin M.H. (2013). Reduction of prostate intrafractional motion from shortening the treatment time. Phys. Med. Biol..

[6] van de Water S., Valli L. (2014). Intrafraction prostate translations and rotations during hypofractionated robotic radiation surgery: Dosimetric impact of correction strategies and margins. Int. J. Radiat. Oncol. Biol. Phys..

[7] Xie Y., Djajaputra D. (2008). Intrafractional motion of the prostate during hypofractionated radiotherapy. Int. J. Radiat. Oncol. Biol. Phys..

[8] Tong X., Chen X. (2015). Intrafractional prostate motion during external beam radiotherapy monitored by a real-time target localization system. J. Appl. Clin. Med. Phys..

[9] Oehler C., Lang S. (2014). PTV margin definition in hypofractionated IGRT of localized prostate cancer using cone beam CT and orthogonal image pairs with fiducial markers. Radiat. Oncol..

[10] Dang A., Kupelian P.A. (2018). Image-guided radiotherapy for prostate cancer. Transl. Androl. Urol..

[11] Curtis W., Khan M. (2013). Relationship of imaging frequency and planning margin to account for intrafraction prostate motion: Analysis based on real-time monitoring data. Int. J. Radiat. Oncol. Biol. Phys..

[12] Winkel D., Bol G.H. (2019). Adaptive radiotherapy: The Elekta Unity MR-linac concept. Clin. Transl. Radiat. Oncol..

[13] Bodusz D., Miszczyk L. (2016). Impact of intrafractional respiratory-induced prostate mobility on PTV size. Tumori.

[14] Ikeda I., Mizowaki T. (2015). Effect of intrafractional prostate motion on simultaneous boost intensity-modulated radiotherapy to the prostate: A simulation study based on intrafractional motion in the prone position. Med. Dosim..

[15] Polat B., Guenther I. (2008). Intra-fractional uncertainties in image-guided intensity-modulated radiotherapy (IMRT) of prostate cancer. Strahlenther. Onkol..

[16] Budiharto T., Slagmolen P. (2011). Intrafractional prostate motion during online image guided intensity-modulated radiotherapy for prostate cancer. Radiother. Oncol..

[17] Thomas S.J., Ashburner M. (2013). Intra-fraction motion of the prostate during treatment with helical tomotherapy. Radiother. Oncol..

[18] Iwama K., Yamazaki H. (2013). Analysis of intrafractional organ motion for patients with prostate cancer using soft tissue matching image-guided intensity-modulated radiation therapy by helical tomotherapy. Anticancer Res..

[19] Kurosawa Y., Ishikawa H. (2012). Intra-fractional set-up and organ motion errors in intensity-modulated radiation therapy for prostate cancer. Nihon Hoshasen Gijutsu Gakkai Zasshi.

[20] Han B., Najafi M. (2018). Evaluation of transperineal ultrasound imaging as a potential solution for target tracking during hypofractionated radiotherapy for prostate cancer. Radiat. Oncol..

[21] Foster R.D., Pistenmaa D.A. (2012). A comparison of radiographic techniques and electromagnetic transponders for localization of the prostate. Radiat. Oncol..

[22] Cetnar A., Ayan A.S. (2021). Prospective dual-surrogate validation study of periodic imaging during treatment for accurately monitoring intrafraction motion of prostate cancer patients. Radiother. Oncol..

[23] Colvill E., Booth J. (2016). A dosimetric comparison of real-time adaptive and non-adaptive radiotherapy: A multi-institutional study encompassing robotic, gimbaled, multileaf collimator and couch tracking. Radiother. Oncol..

[24] Wilbert J., Baier K. (2013). Accuracy of real-time couch tracking during 3-dimensional conformal radiation therapy, intensity modulated radiation therapy, and volumetric modulated arc therapy for prostate cancer. Int. J. Radiat. Oncol. Biol. Phys..

[25] Lovelock D.M., Messineo A.P. (2015). Continuous monitoring and intrafraction target position correction during treatment improves target coverage for patients undergoing SBRT prostate therapy. Int. J. Radiat. Oncol. Biol. Phys..

[26] de Muinck Keizer D.M., Kontaxis C. (2020). Dosimetric impact of soft-tissue based intrafraction motion from 3D cine-MR in prostate SBRT. Phys. Med. Biol..

[27] Dang T.M., Peters M.J. (2017). Efficacy of flattening-filter-free beam in stereotactic body radiation therapy planning and treatment: A systematic review with meta-analysis. J. Med. Imaging Radiat. Oncol..

[28] de Leon J., Jameson M.G. (2019). Reduced motion and improved rectal dosimetry through endorectal immobilization for prostate stereotactic body radiotherapy. Br. J. Radiol..

[29] Jones B.L., Gan G. (2012). Dosimetric and deformation effects of image-guided interventions during stereotactic body radiation therapy of the prostate using an endorectal balloon. Med. Phys..

[30] Hedrick S.G., Fagundes M. (2017). A comparison between hydrogel spacer and endorectal balloon: An analysis of intrafraction prostate motion during proton therapy. J. Appl. Clin. Med. Phys..

[31] Tetar S., Bruynzeel A. (2019). Clinical implementation of magnetic resonance Imaging guided adaptive radiotherapy for localized prostate cancer. Phys. Imaging Radiat. Oncol..

